# Flexible Cognitive Strategies during Motor Learning

**DOI:** 10.1371/journal.pcbi.1001096

**Published:** 2011-03-03

**Authors:** Jordan A. Taylor, Richard B. Ivry

**Affiliations:** 1 Department of Psychology, University of California, Berkeley, Berkeley, California, United States of America; 2 Helen Wills Neuroscience Institute, University of California, Berkeley, Berkeley, California, United States of America; University College London, United Kingdom

## Abstract

Visuomotor rotation tasks have proven to be a powerful tool to study adaptation of the motor system. While adaptation in such tasks is seemingly automatic and incremental, participants may gain knowledge of the perturbation and invoke a compensatory strategy. When provided with an explicit strategy to counteract a rotation, participants are initially very accurate, even without on-line feedback. Surprisingly, with further testing, the angle of their reaching movements drifts in the direction of the strategy, producing an increase in endpoint errors. This drift is attributed to the gradual adaptation of an internal model that operates independently from the strategy, even at the cost of task accuracy. Here we identify constraints that influence this process, allowing us to explore models of the interaction between strategic and implicit changes during visuomotor adaptation. When the adaptation phase was extended, participants eventually modified their strategy to offset the rise in endpoint errors. Moreover, when we removed visual markers that provided external landmarks to support a strategy, the degree of drift was sharply attenuated. These effects are accounted for by a setpoint state-space model in which a strategy is flexibly adjusted to offset performance errors arising from the implicit adaptation of an internal model. More generally, these results suggest that strategic processes may operate in many studies of visuomotor adaptation, with participants arriving at a synergy between a strategic plan and the effects of sensorimotor adaptation.

## Author Summary

Motor learning has been modeled as an implicit process in which an error, signaling the difference between the predicted and actual outcome is used to modify a model of the actor-environment interaction. This process is assumed to operate automatically and implicitly. However, people can employ cognitive strategies to improve performance. It has recently been shown that when implicit and explicit processes are put in opposition, the operation of motor learning mechanisms will offset the advantages conferred by a strategy and eventually, performance deteriorates. We present a computational model of the interplay of these processes. A key insight of the model is that implicit and explicit learning mechanisms operate on different error signals. Consistent with previous models of sensorimotor adaptation, implicit learning is driven by an error reflecting the difference between the predicted and actual feedback for that movement. In contrast, explicit learning is driven by an error based on the difference between the feedback and target location of the movement, a signal that directly reflects task performance. Empirically, we demonstrate constraints on these two error signals. Taken together, the modeling and empirical results suggest that the benefits of a cognitive strategy may lie hidden in many motor learning tasks.

## Introduction

When learning a new motor skill, verbal instruction often proves useful to hasten the learning process. For example, a new driver is instructed on the sequence of steps required to change gears when using a standard transmission. As the skill becomes consolidated, the driver no longer requires explicit reference to these instructions. Operating a vehicle with a stiffer or looser clutch does not generally require further instruction, but rather entails a subtle recalibration, or adaptation of the previously learned skill. Indeed, the use of an explicit strategy may even lead to degradation in the expert's performance. Consideration of these contradictory issues brings into question the role of instructions or explicit strategies in sensorimotor learning.

The type of motor task and nature of the instruction can have varying effects on motor execution and learning [1]–[3]. In the serial reaction time task (SRT), participants produce a sequence of cued button presses. If the participant is informed of the underlying sequence, learning occurs much more rapidly compared to when sequential learning arises from repeated performance [4]. However, learning in the SRT task entails the linkage of a series of discrete actions. Explicit instructions of the sequence structure may be viewed as a way to create a working memory representation of the series. Many skills lack such a clear elemental partition and, as such, participants cannot easily verbalize what a successful movement entails. For example, the pattern of forces required to move the hand in a straight line in a novel force field [5]–[7] would be hard to verbalize.

Various studies have examined the role of explicit strategies in tasks involving sensorimotor adaptation [8]–[11]. The benefits of an explicit strategy may be illusory with adaptive processes arising from automatic and incremental updating of a motor system that is impenetrable to conscious intervention [12]–[16]. However, performance measures indicate that adaptation may differ between conditions in which participants are either aware or unaware of the changes in the environment [17]. For example, a large visuomotor rotation can be introduced abruptly, in which case, awareness is likely, or introduced incrementally such that participants are unaware of the rotation. The abrupt onset of large unexpected errors may promote the use of cognitive strategies [18]–[20]. Participants who gain explicit knowledge of an imposed visuomotor rotation show better performance during learning than participants who report little or no awareness of the rotation [10]. Moreover, the rate of learning, at least in the early phase of adaptation, correlates positively with spatial working memory span [21], suggesting that strategic compensation may be dependent on working memory capacity. Studies of sensorimotor adaptation during aging also indicate that the rate of learning is slower in older adults compared to young adults, despite similar aftereffects [22]–[24]. This cost is absent in older adults who report awareness of the rotation [25].

In many of the studies cited above, the assumption has been that the development of awareness can lead to the utilization of compensatory strategies. However, few studies have directly sought to manipulate strategic control during sensorimotor adaptation. One striking exception is a study by Mazzoni and Krakauer [9]. Participants viewed a display of eight small circles, or visual landmarks, that were evenly spaced by 45° to form a large, implicit ring. The target location was specified by presenting a bullseye within one of the eight circles. After an initial training phase in which the visuomotor mapping was unaltered, a 45° rotation in the counterclockwise direction (CCW) was introduced. In the standard condition in which no instructions were provided, participants gradually reduced endpoint error by altering their movement heading in the clockwise direction (CW). In the strategy condition, participants were given explicit instructions to move to the circle located 45° clockwise to the target. This strategy enabled these participants to immediately eliminate all error. However, as training continued, the participants progressively increased their movement heading in the clockwise direction. As such, the endpoint location of the feedback cursor drifted further from the actual target location and, thus, performance showed an increase in error over training, a rather counterintuitive result [26].

Mazzoni and Krakauer [9] proposed that this drift arises from the implicit adaptation of an internal forward model. Importantly, the error signal for this learning process is not based on difference between the observed visual feedback and target location. Rather, it is based on the difference between the observed visual feedback and strategic aiming location. Even though participants aim to a clockwise location of the target (as instructed), the motor system experiences a mismatch between the predicted state and the visual feedback. This mismatch defines an error signal that is used to recalibrate the internal model. Reducing the mismatch results in an adjustment of the internal model such that the next movement will be even further in the clockwise direction. Thus, the operation of an implicit learning process that is impervious to the strategy produces the paradoxical deterioration in performance over time.

In the present paper, we start by asking how this hypothesis could be formalized in a computational model of motor learning. State space modeling techniques have successfully described adaptation and generalization during motor learning [27]–[29]. These models focus on how learning mechanisms minimize error from trial to trial. Variants of these models postulate multiple learning mechanisms that operate at different time scales [28]. Within this framework, strategic factors might be associated with fast learning processes that rapidly reduce error. However, such models are unable to account for the drift that arises following the deployment of a strategy. To address these issues, we developed a series of setpoint state-space models of adaptation to quantitatively explore how strategic control and implicit adaptation interact. Assuming a fixed strategy, adaptation should continue to occur until the error signal, the difference between the feedback location and the aiming location is zero; that is, the visual feedback matches the intended aim of the reach. As such, drift arising from implicit adaptation should continue to rise until it offsets the adopted strategy. To test this prediction, we increased the length of the adaptation phase. Moreover, we manipulated the salience of the visual landmarks used to support the strategy. We hypothesized that these landmarks served as a proxy for the aiming location. If this assumption is correct, then elimination of the visual landmarks should weaken the error signal, given uncertainty concerning the aiming location, and drift should be attenuated. We test this prediction by comparing performance with and without visual landmarks.

## Results

**Figure 1:**
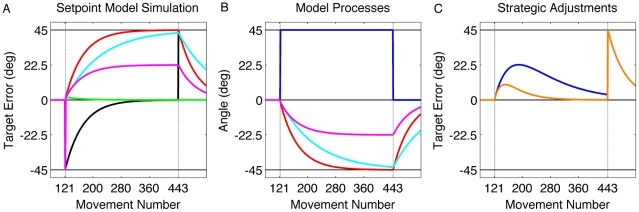
Setpoint model simulation. A −45° rotation was introduced on movement 121 and remained present for the next 322 trials. A) Simulated target error for four state space models. Black: Standard state-space model (A = 1, B = 0.02); Green: Setpoint model in which the target error is used to adapt the internal model; Red: Setpoint model with direct-feedthrough in which the aiming error is used to adapt the internal model. Drift is attenuated by either reduced adaptation rate (Cyan: A = 1, B = 0.01) or reducing the availability of the aiming error signal that selectively operates on the strategy (Magneta: K = 0.5). B) Internal model estimation of rotation when a fixed strategy (blue) is combined with either high (red, K = 1) or low (magenta, K = 0.5) certainty of aiming errors. C) Effect of a variable strategy on target error. The strategy was simulated with a low (blue; E = 1 and F = 0.01) or high (orange; F = 0.05) weighting of the target error.

Current models of sensorimotor adaptation have not addressed the effect of explicit strategies. Therefore, we started with the standard state-space model (Eq 1 and 2), and incrementally modified it to accommodate the use of an explicit strategy. The standard model for target error is given as:
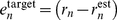
(1)

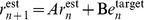
(2)where 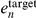 is the target endpoint error on movement n, 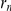
is the rotation, 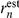
is the internal model's estimation of the rotation, A is a memory term, and B represents either a learning rate or sensitivity to error [27]–[30]. As expected, this model gradually learns to compensate for a visuomotor rotation (Figure 1A – black line; simulated with A  = 1 and B  = 0.02).

### Modeling strategy use during visuomotor adaptation

When informed of an appropriate strategy that will compensate for the rotation, participants immediately counteract the rotation and show on-target accuracy. The standard model as formulated above does not provide a mechanism to implement an explicit strategy. To allow immediate implementation of the strategy, we postulate that there is direct feedthrough of the strategy (s) to the target error equation (equation 1):
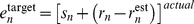
(3)


Direct feedthrough allows the strategy to contribute to the target error equation without directly influencing the updating of the internal model. If the strategy operated through the internal model, then the impact of the strategy would take time to evolve, assuming there is substantial memory of the internal model's estimation of the rotation (i.e., A has a high value in Eq. 2). With direct feedthrough, the implementation of an appropriate strategy can immediately compensate for the rotation. In the current arrangement, the appropriate strategy is fixed at 45° in the CW direction from the cued target.

**Figure 2:**
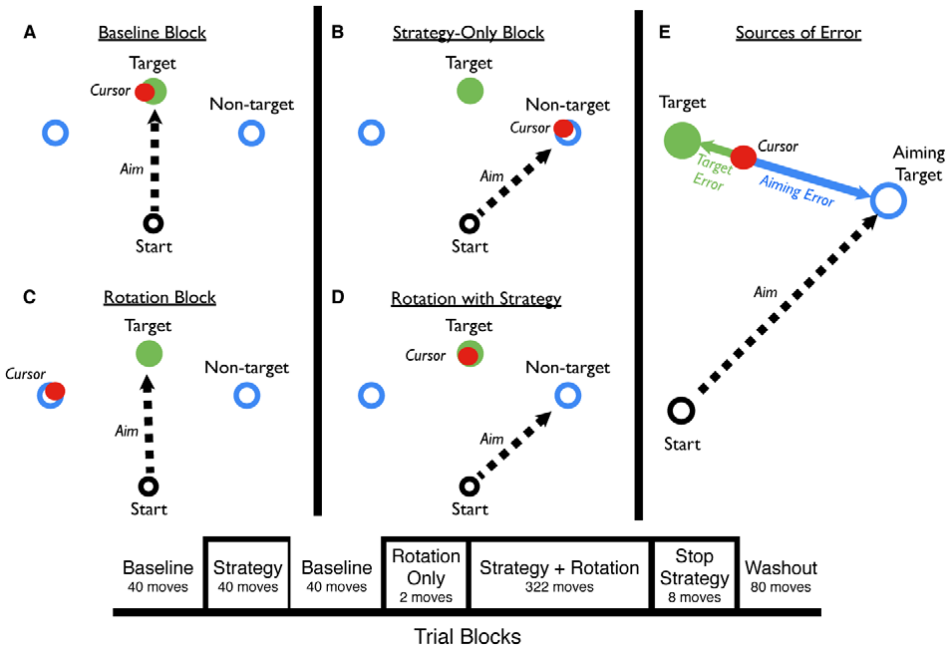
Experimental task design. The experiment workspace consisted of 8 empty blue circles separated by 45° (three locations are shown here). The target was defined when a green circle appeared at one of the locations. The hand was occluded by the apparatus and on feedback trials, a red cursor appeared as soon as the participant crossed a virtual ring, 10-cm from the start location. A) In the baseline block, participants moved towards the cued green target. B) In the strategy-only block, participants moved to the blue circle located 45° in the clockwise direction. Feedback was presented at the veridical hand position. C) For the two rotation probes, participants were instructed to move to the green target, but feedback of hand position was rotated 45° in the counter-clockwise direction. D) In the rotation plus strategy block, participants were instructed to move to the blue circle located 45° clockwise direction from the target. The feedback of hand position was rotated 45° counter-clockwise. E) Two sources of movement error: a target error between the feedback location and target location and an aiming error between the feedback location and aiming location.



Once the strategy is implemented, performance should remain stable since the error term is small. Indeed, a model based on Eq. 3 immediately compensates for the rotation. The target error, the difference between the feedback location and target location, is essentially zero on the first trial with the strategy, and remains so throughout the rotation block (Figure 1B – green line). However, this model fails to match the empirical results observed by Mazzoni and Krakauer [9]: performance drifts over time with an increase in errors in the direction of the strategy. This phenomenon led the authors to suggest that the prediction error signal to the internal model is not based on target error. Instead, the error signal should be defined by the difference between the feedback location and aiming location (see Figure 2E):

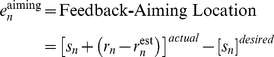
(4)The formulation of the prediction error term in Eq. 4 is akin to a setpoint or reference signal from engineering control theory [30]. In typical motor learning studies, the setpoint is to reach to the target. When there is no strategy (s = 0), the target error in Eq. 1 is the same as the error term in Eq. 4. However, when a strategy with direct feedthrough is used (s≠0), the strategy terms may cancel out if the actual implemented strategy is similar to the desired strategy. The input error to update the internal model's estimate of the rotation becomes:
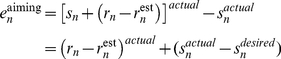
(5)


This model shows immediate compensation for the visuomotor rotation, and more importantly, produces a gradual deterioration in performance over the course of continued training with the reaching error drifting in the direction of the strategy (Figure 1A – red line), consistent with the results reported by Mazzoni and Krakauer [9].

It is important to emphasize that the error signal for sensorimotor recalibration in Eq. 4 is not based on the difference between the feedback location and target location (target error). Rather, the error signal is defined by the difference between the feedback location and aiming location, or what we will refer to as aiming error. When a fixed strategy is adopted throughout training (Figure 1B – blue line), the aiming error is (initially) quite large given that the predicted hand location is far from the location of visual feedback, even though the feedback cursor may be close to the actual target. In its simplest form, the setpoint model predicts that, as the internal model minimizes this error (Figure 1B – red line), drift will continue until the observed feedback of the hand matches the aiming location. That is, the magnitude of the drift should equal the size of the strategic adjustment. In the Mazzoni and Krakauer set-up [9], the drift would eventually reach 45° in the CW direction (Figure 1A – red line).

A second prediction can be derived by considering that the error signal in Eq. 4 relies on an accurate estimate of the strategic aiming location. We assume that a visual landmark in the display can be used as a reference point for strategy implementation (e.g., the blue circle adjacent to the target). This landmark can serve as a proxy for the aiming location. The salience of this landmark provides an accurate estimate of the aiming location and, from Eq. 4, drift should be pronounced. However, if these landmarks are not available, then the estimate of the aiming location will be less certain. Previous studies have shown that adaptation is attenuated when sensory feedback is noisy [31], [32]. One approach for modeling the effect of changing the availability or certainty of the (strategy defined) aiming location would be to vary the adaptation rate (B). For example, B could be smaller if there is a decrease in certainty of the aiming location, and correspondingly, a decrease in the certainty of the aiming error. This model predicts that the rate of drift is directly related to B: if B is lower due to decreased certainty of the aiming location, then the rate of drift will be attenuated (Figure 1A – cyan line).

To evaluate the predictions of this setpoint model, participants were tested in an extended visuomotor rotation task in which we varied the visual displays used to define the target and strategic landmarks (see methods). The target was defined as a green circle, appearing at one of eight possible locations, separated by 45° (Figure 2, only three shown here). By encouraging the participants to make movements that “sliced” through the target, and only providing feedback at the point of intersection with the virtual target ring, we were able to train the participants to move quickly with relatively low trial-to-trial variability. We assume that participants mostly relied on feedforward control given the ballistic nature of the movements and absence of continuous online feedback.

Participants were assigned to one of three experimental groups (n = 10 per group), with the groups defined by our manipulation of the blue landmarks in the visual displays. For the aiming-target group (AT), the blue circles were always visible, similar to the method used by Mazzoni and Krakauer. For the disappearing aiming-target group (AT), the blue circles were visible at the start of the trial and disappeared when the movement was initiated. For the no aiming-target group (NoAT), the blue landmarks were not included in the display.

**Figure 3:**
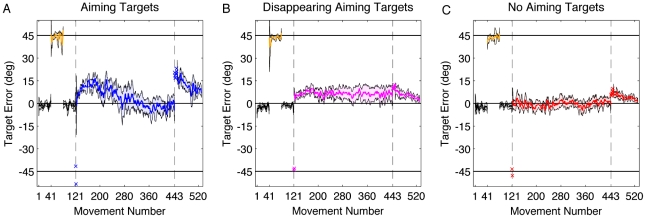
Group averaged endpoint error relative to the target for the three groups. Participants first practiced moving to the cued target without a rotation (black) and while using the strategy without a rotation (orange). The rotation was turned on between movements 121 and 443 (dashed vertical lines). For the first two of these trials, the rotation probes, the participants had not been given the strategy (X's). For the next 320 rotation trials, participants were instructed to use the strategy. Following this, the rotation was turned off and participants were instructed to move towards the cued target, first without endpoint feedback (X's) and then with endpoint feedback (circles). A) Aiming-Target Group (blue). B) Disappearing Aiming-Target Group (magenta). C) No Aiming-Target Group (red). Shading represents the 95% confidence interval of the mean.

The participants were initially required to reach to the green target (Figure 2A). Movement duration, measured when the hand crossed the target ring, averaged 275±50.8 ms with no significant difference between groups (F_2,27_ = 1.02, p = 0.37). Following the initial familiarization block, participants were trained to use a strategy of moving 45° in the CW direction from the green target location, (Figure 2B). This location corresponded to the position of the neighboring blue circle. Feedback was veridical in this phase (e.g., corresponded to hand position). To help participants in the NoAT group learn to move at 45°, the blue circles were also presented on half of the trials for this group (in this phase only). The mean angular shifts, relative to the green target, were 43.4±1.6° and 42.9±1.2° for the AT and DAT groups, respectively (Figure 3 - orange). For the NoAT group, the mean angular shift was 43.5±0.9° when the aiming target was present and 40.1±7.1° when the aiming target was absent. While the variance was considerably larger for trials without the aiming target, the means were not significantly different (t_18_ = 0.95, p = 0.38).

Practicing the 45° CW strategy did not produce interference on a subsequent baseline block in which participants were again instructed to reach to the cued, green target (Figure 3 – black). Over the last 10 movements of the familiarization block participants, across all groups, had an average target error of −1.5±0.7°. Over the first 10 movements of the baseline block, this value was −0.5±0.6°, confirming that the strategy-only block did not produce a substantial bias.

Without warning, the CCW rotation was introduced (Figure 2C). As expected, the introduction of the CCW rotation induced a large target error. Averaged over the two, rotation probe trials, the mean values were −41.6±3.3°, −43.8±1.1°, −43.5±3.2° for AT, DAT, and NoAT groups, respectively (Figure 3 – “x”). After the participants were instructed to use the clockwise strategy (Figure 2D), the target error was reduced immediately to 3.5±4.4°, 1.0±4.3°, and −2.5±6.6°, values that were not significantly different from each other (F_2,27_ = 1.96, p = 0.16).

**Figure 4:**
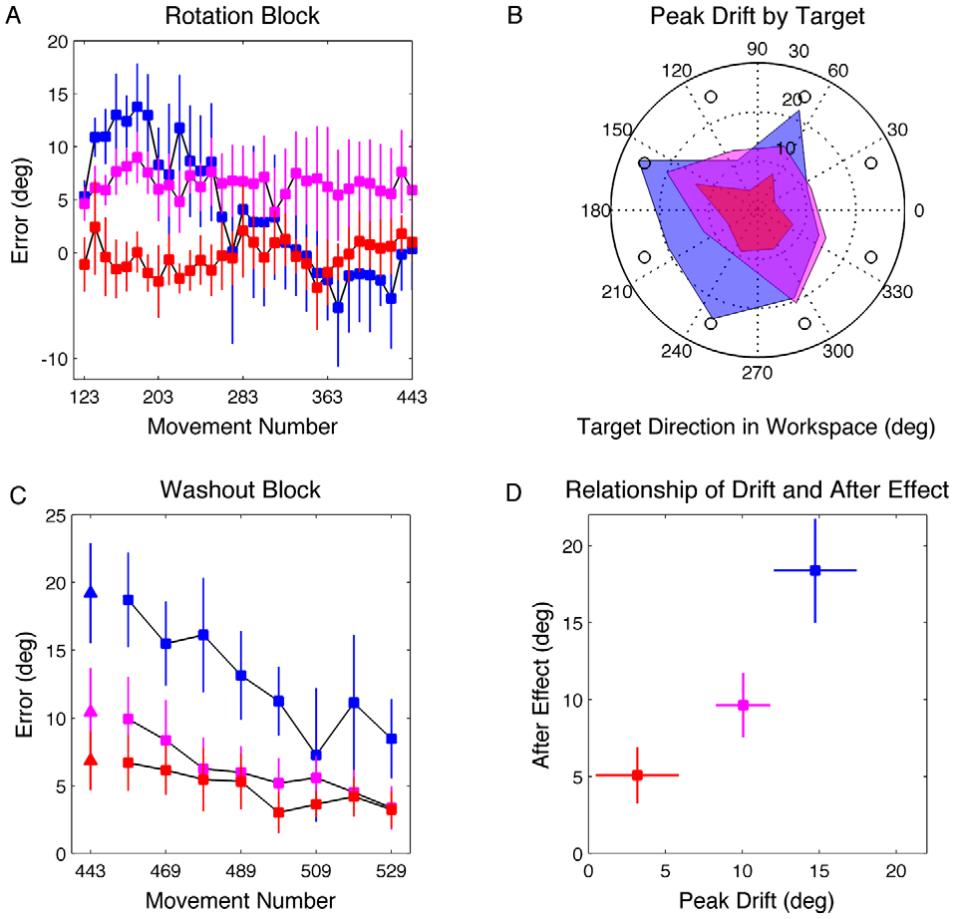
Time course of drift and aftereffect, and the relationship of drift to target location and aftereffect. A) Average endpoint angular error relative to the target for the three groups, binned by averaging over epochs of ten movements (AT group in blue, DAT group in magenta, NoAT group in red). B) Peak drift with respect to the eight target location for the three groups. The empty circles are the target locations. To identify peak drift, 10 bins of four movements were calculated for each direction. C) Angular error after the rotation was turned off and participants were instructed to stop using the strategy. Triangles are average of the first eight post-rotation trials, performed without visual feedback. Squares are washout block with feedback. D) Relationship of drift and aftereffect based on the estimated peak drift for each participant and the first eight post-rotation trials. For B) and D), the means and 95% confidence interval of the mean were estimated through bootstrapping.

The participants were then instructed to use the strategy and required to produce a total of 320 reaching movements under the CCW rotation. This extended phase allowed us to a) verify that error increased over time, drifting in the direction of the strategy, and b) determine if the magnitude of the drift would approximate the magnitude of the rotation, a prediction of the simplest form of the setpoint model. Consistent with the results of Mazzoni and Krakauer [9], error increased in the direction of the strategy over the initial phase of the rotation block. However, the extent of the drift fell far short of the magnitude of the rotation. To quantify the peak drift, each participant's time series of endpoint errors was averaged over 10 movements and we identified the bin with the largest error. Based on this estimate of peak drift, a significant difference was observed between groups (F_2,27_ = 21.9, p<0.001; Figure 4A). This is consistent with the prediction of the model based that the salience of the aiming targets would influence the estimation of the aiming location. Drift was largest when the aiming targets were always visible, and progressively less for the DAT and NoAT groups. Drift was not isolated to particular target locations (Figure 4B).

**Figure 5:**
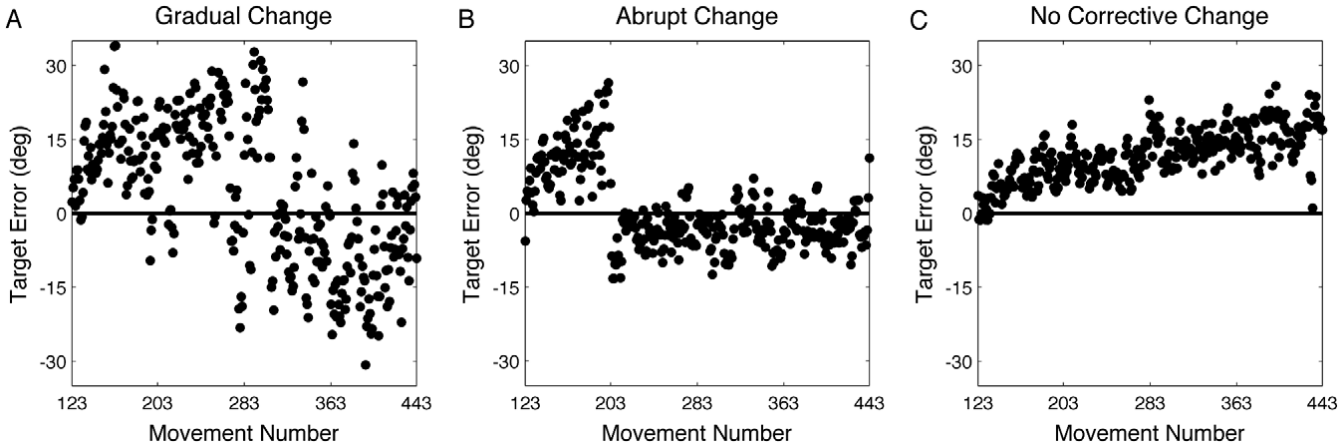
Performance during the rotation block of three participants. A and B are from the AT group; C is from the DAT group. A) Drift followed by large fluctuations in error. B) Drift followed by an abrupt change in target error. C) Continuous drift across training.

Our rotation plus strategy block lasted 320 trials, nearly four times the number of trials used by Mazzoni and Krakauer [9]. This larger window provides an interesting probe on learning given that the participants become progressively worse in performance with respect to the target over the drift phase. While the AT group had the largest drift, they eventually showed a change in performance such that the heading angle at the end of the rotation block was close to 45° CW from the green target (Figure 3A). By the end of training, their target error was only 0.3±3.9°, which was not significantly different from zero (t_9_ = 0.17, p = 0.85). We did not observe a consistent pattern in how these participants counteracted the drift (Figure 5). Two participants showed clear evidence of an abrupt change in their performance, suggesting a discrete change in their aiming strategy. For the other eight AT participants, the changes in performance were more gradual.

The drift persisted over the 320 trials of the rotation block for participants in the DAT group (Figure 3C). The average drift was 5.9±4.8° at the end of training, a value that was significantly greater than zero (t_9_ = 2.40, p = 0.04). Given that the NoAT group showed minimal drift, we did not observe any consistent changes in performance over the block. At the end of training, the mean target error was only 1.0±1.9°, a value which is not significantly different from zero (t_9_ = 1.01, p = 0.33).

### The effect of aiming target availability

The availability or certainty in the estimate of the aiming location was manipulated by altering the presence of the aiming target across the groups. As predicted by the setpoint model, the degree of drift was attenuated as the availability of the aiming targets decreased. In the current implementation of our model, this decrease in drift rate is captured by a decrease in the adaptation rate (B): with greater uncertainty, the weight given to the error term for updating the internal model is reduced.

However, one prediction of this model is at odds with the empirical results. Variation in the adaptation rate not only predicts a change in drift rate, but also predicts a change in the washout period. Specifically, decreasing the adaptation rate should produce a slower washout, or extended aftereffect (Figure 1B – cyan). This prediction was not supported. The washout rates are similar across the three groups (bootstrap, p>0.11 between all groups). One could hypothesize different adaptation rates during the rotation and washout phases, with the effect of target certainty only relevant for the former. However, a *post hoc* hypothesis along these lines is hard to justify.

Alternatively, it is possible that the adaptation rate (B) is similar for the three groups and that the variation in drift rate arises from another process. One possibility is that the manipulation of the availability of the aiming targets influences the certainty of the desired strategy term in Equation 4, and correspondingly, modifies the aiming error term:
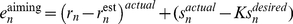
(6)


A value of K that is less than 1 will attenuate drift (Figure 1A – magenta line; simulated with K = 0.5) because the strategy output (Eq. 3) and the desired strategy (Eq. 6) do not completely cancel out. Consequently, the error used to adjust the internal model will be smaller and produce attenuated drift (Figure 1B – magenta line). Moreover, because the strategy is no longer used during the washout phase, the K term is no longer relevant. Thus, the washout rates should be identical across the three groups, assuming a constant value of B.

In sum, while variation in B or K can capture the group differences in drift rate, only the latter accounts for the similar rates of washout observed across groups. When the availability of the aiming targets is reduced, either by flashing them briefly or eliminating them entirely, the participants' certainty of the aiming location is attenuated. This hypothesis is consistent with the notion that the aiming locations serve as a proxy for the predicted aiming location.

### Strategy adjustment based on performance error

As noted above, none of the participants showed drift approaching 45°. Even those exhibiting the largest drift eventually reversed direction such that they became more accurate over time in terms of reducing endpoint error with respect to the target location. To capture this feature of the results, we considered how participants might vary their strategy over time as performance deteriorates. It is reasonable to assume that the participant may recognize that the adopted strategy should be modified to offset the rising error. One salient signal that could be used to adjust the strategy is the target error, the difference between the target location and the visual feedback.

To capture this idea, we modified the setpoint model, setting the strategy as a function of target error (Figure 2E):
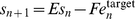
(7)where E defines the retention of the state of the strategy and the F defines the rate of strategic adjustment. As target error grows (i.e., drift), the strategy will be adjusted to minimize this error (Figure 2E). In our initial implementation of the setpoint model, the strategy term was fixed at 45°. Equation 7 allows the strategy term to vary, taking on any value between 0° and 360°.

The availability of the aiming targets, captured by K in Eq. 6, influences the magnitude of the drift. Greater drift occurs when the aiming error, that between the feedback location and aiming location, is salient (Figure 1B – red line; K = 1). However, when the target error grows too large, adjustments to the strategy begin to gain momentum and performance becomes more accurate with respect to the target given the change in strategy (Figure 1C – blue line; simulated with E = 1 and F = 0.01). More emphasis on target errors rather than the aiming error results in less drift (Figure 1C – orange line; simulated with F = 0.05). Thus, the relative values of K and F determine the degree of performance error that is tolerated before strategic adjustments compensate to offset the drift (Figure 1C).

This setpoint model (Eqs. 8–11) was fit by bootstrapping (see methods) each group's time series of target errors:
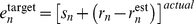
(8)

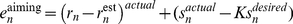
(9)

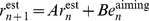
(10)

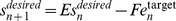
(11)


**Figure 6:**
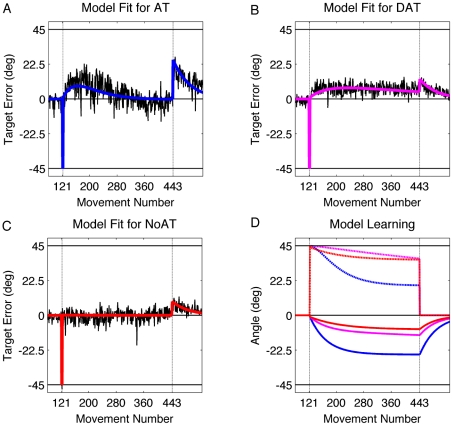
Setpoint model fit. The setpoint model with direct-feedthrough (equations 8–11) was fit to each group's data (from figure 3) via bootstrapping. A–C: Solid functions are averaged model fits for the AT group (blue), DAT group (magenta), and NoAT group (red) in comparison with actual group averaged data (black). D) The target error is the combination of learning within the sensorimotor recalibration process (solid) and the aiming location associated with the strategy (dashed), shown here fro the AT group (blue), DAT group (magenta), and NoAT group (red).

The fits (Figure 6A–C and Table 1) show that K is the greatest for the AT group and progressively less for the DAT group and the NoAT group (AT vs DAT group: p = 0.003; AT vs NoAT groups: p<0.001; DAT vs. NoAT groups: p<0.001). When the aiming targets remain visible, the aiming error signal is readily available, and the weight given to the strategic aiming location, K, is larger. Conversely, the weight given to the target error, F, is significantly greater for the NoAT group compared to the AT and DAT groups (NoAT vs AT group: p = 0.005; NoAT vs DAT group p = 0.001). These results are consistent with the hypothesis that participants in the NoAT group rely more on target errors because the absence of the aiming targets removes a reference point for generating a reliable aiming error (Eq. 9).

**Table 1:** Modeling results for each group based on the setpoint model (Eqns 8–11).

	AT Group	DAT Group	NoAT Group
**A**		0.991±0.002	
**B**		0.012±0.003	
**E**		0.999±0.001	
**K**	0.985±0.034	0.409±0.122	0.195±0.108
**F**	0.023±0.006	0.002±0.003	0.725±0.319
**Goodness of fit (r)**	0.682±0.075	0.713±0.069	0.650±0.081

A: Retention factor of the internal model; B: Adaptation rate based upon aiming errors; E: Retention factor of the strategy; K: Availability of the strategic aiming location; F: Adjustment rate of the strategy based upon target errors (high values favor strategy change). A, B, and E were constrained to be the same across groups, while parameters K and F were allowed to vary between groups. The means and 95% confidence interval of the mean were estimated through bootstrapping.

The dynamics of the recalibration process and strategy state (Eqs. 10 and 11) are plotted in Figure 6D. These parameters, along with the other parameters that represent the memory of the internal model (A), the adaptation rate (B), and the memory of the strategy (E) are listed in Table 1.

Following the rotation block, we instructed the participants that the rotation would be turned off and they should reach to the cued green target. For the first eight trials, no endpoint feedback was presented. This provided a measure of the degree of sensorimotor recalibration in the absence of learning (Figure 4C – triangles). Aftereffects were observed in all three groups. The average error was significantly different from zero in the CW direction from the green target for all three groups (one sample t-test for each group, p<0.001). In comparisons between the groups, the AT group showed the largest aftereffect of 19.2±3.7° (t_18_ = 3.5, p = 0.003 and t_18_ = 5.61, p<0.001 compared to the DAT and NoAT groups, respectively). The mean aftereffects for the DAT and NoAT groups were 10.4±3.2° and 6.8±2.2°, values that were not significantly different. When endpoint feedback was again provided, the size of the aftereffect diminished over the course of the washout block (Figure 4C - squares).

### Relationship between drift and aftereffect

In the setpoint model, the internal model will continue to adapt even in the face of strategic adjustments adopted to improve endpoint accuracy. As such, the model predicts that the size of the aftereffect should be larger than the degree of drift. To test this prediction, we compared the peak drift during the rotation block to the aftereffect. In the preceding analysis, we had estimated peak drift for each participant by averaging over 10 movements and identifying the bin with the largest error. However, a few errant movements could easily bias the estimate of drift within a 10-movement bin. As an alternative procedure, we used a bootstrapping procedure to identify the bin with the largest angular error for each group. This method should decrease the effect of noise because the estimate of peak drift is selected from an averaged sample of the participants' data. Moreover, any bias in the estimate of the magnitude of the peak should be uniform across the three groups of participants. For consistency, we estimated the aftereffect (the first 8 trials without feedback) using the same bootstrap procedure.

For the AT group, the peak drift was 14.8±2.5° in the CW direction, occurring 64±30 movements into the rotation block. For the DAT group, the peak drift was 10.0±1.8°, occurring at a later point in the rotation block (130±106). For the NoAT group, peak drift was only 3.2±2.7° and occurred after 145±131 movements. As predicted by the model, the aftereffect was significantly larger than peak drift for the AT and NoAT groups (Figure 4D; bootstrap: p = 0.002 and p<0.001, respectively). The difference between the degree of peak drift and aftereffect in the DAT group was not reliable. It is important to emphasize that estimates of the time of peak drift should be viewed cautiously, especially in terms of comparisons between the three groups. These estimates have lower variance for the AT group because it was easier to detect the point of peak drift in this group compared to the DAT and NoAT groups.

## Discussion

### Behavioral summary

Visuomotor rotation tasks are well-suited to explore how explicit cognitive strategies influence sensorimotor adaptation. Following the approach introduced by Mazzoni and Krakauer [9], we instructed participants to aim 45° CW in order to offset a −45° rotation. Between groups, we manipulated the information available to support the strategy by either constantly providing an aiming target, blanking the aiming target at movement initiation, or never providing an aiming target. In all groups, the strategy was initially effective, resulting in the rapid elimination of the rotation-induced endpoint error. However, when the aiming target was present, participants showed a drift in the direction of the strategy, replicating the behavior observed in Mazzoni and Krakauer [9]. This effect was markedly attenuated when the aiming target was not present suggesting that an accurate estimate of the strategic aiming location is responsible for causing the drift. In addition, when the drift became quite large (as in the AT group), participants begin to adjust their strategy to offset the implicit drift.

### Incorporating a strategy into state-space models

Mathematical models of sensorimotor adaptation have not explicitly addressed how a strategy influences learning and performance. By formalizing the effect of strategy usage into the standard state-space model of motor learning, we can begin to evaluate qualitative hypotheses that have been offered to account for the influence of strategies on motor learning. Mazzoni and Krakauer [9] suggested that drift reflects the interaction of the independent contribution of strategic and implicit learning processes in movement execution. Current models of adaptation cannot be readily modified to account for this interaction. Rather, we had to consider more substantive architectural changes. Borrowing from engineering control theory, we used a setpoint model in which the internal model can be recalibrated around any given reach location. The idea of a setpoint is generally implicit in most models of learning, but this component does not come into play since the regression is around zero. However, simply making the setpoint explicit is not sufficient to capture the drift phenomenon. The strategy must have direct feedthrough to the output equation in order to implement the explicit strategy while allowing for an internal model to implicitly learn the visuomotor rotation.

This simple setpoint model was capable of completely eliminating error on the first trial and capture the deterioration of performance with increased training. Drift arises because the error signal is driven by the difference between the internal model's prediction of the aiming location and the actual, endpoint feedback. The idea that an aiming error signal is the source of drift is consistent with the conjecture of Mazzoni and Krakauer [9]. An important observation in the current study is that, given uncertainty in the prediction of the aiming location, participants use external cues as a proxy in generating this prediction. This hypothesis accounts for the observation that drift was largest when the aiming target was always visible, intermediate when the aiming target was only visible at the start of the trial, and negligible when the aiming target was never visible. The aiming target, when present, served as a proxy for predicted hand position, and helped define the error between the feedback cursor and aiming location in visual coordinates. When the aiming target was not present, the aiming location was less well-defined in visual coordinates, and thus, the relationship between the aiming location and feedback cursor was less certain. Under this condition, the participant's certainty of the error was reduced and adaptation based of this signal was attenuated. Quantitatively, progressively smaller values of K were observed with decreasing availability of the aiming targets.

The attenuation of adaptation with increasing uncertainty (as reflected by reduced drift) is similar to the effects on adaptation predicted by a Kalman filter when measurement noise is large. Several studies have shown that adaptation rates can change when the certainty of sensory information is manipulated [31], [32]. In our study, variation in certainty of the desired aiming location (K) influenced the magnitude of drift. As the availability of the aiming targets was reduced, the corresponding estimate of the aiming error became less certain, producing slower adaptation of the internal model, or reduced drift. Moreover, since K directly operates on the estimate of the strategic aiming location, this parameter does not affect the rate of washout since the strategy is no longer used. Consistent with this prediction, the rate of washout was similar across the three groups.

The effect of the visual landmarks on adaptation also provides insight into why other studies have not observed drift, even when participants develop some explicit awareness of the rotation, and presumably, use that knowledge [8], [10], [11], [24], [25] to improve performance rapidly. Several key methodological differences are relevant. First, in most visuomotor rotation studies, online visual feedback is provided during the movements. This may impede drift because participants observe the casual relationship between movement of their hand and the endpoint, cursor feedback [33]. Drift itself could be corrected by online feedback. Second, participants in the earlier studies were not given a clear, explicit strategy, and importantly, were not provided with visual landmarks that could support a self-generated strategy. Under such conditions, participants face a difficult estimation process. The absence of landmarks would increase uncertainty in implementing a self-generated strategy. Moreover, the motor system would not have a salient visual signal for grounding the comparison of feedback and aiming location. As shown by our no-aiming target condition, drift is minimal when the landmarks are absent. Thus, the absence of drift in the visuomotor adaptation literature cannot be taken as evidence that strategies are not relevant. It is likely that, when initial error signals are large, learning involves a combination of strategic and recalibration processes.

### Two sources of errors

Our model entails two types of error signals: an aiming prediction error between the feedback location and aiming location, and performance error between the feedback location and the target location (Figure 2E). The aiming error drives the drift phenomenon while the target error is used to restore performance. Intuitively, the motor system should be able to recalibrate the internal model around any desired reach location, a feature captured by the setpoint model. When there is an accurate estimate of the strategy (the setpoint), then the strategy naturally falls out of the error equation, allowing the internal model to recalibrate around any position. The setpoint mechanism is revealed when a strategy is imposed to counteract a visuomotor rotation. A counterintuitive consequence of this process is the rise in error over time because the motor system is recalibrating around the strategic aiming location (or its proxy) and not the target location.

Interestingly, while there was an initial rise in endpoint error, this function eventually reversed, returning close to zero endpoint error by the end of the strategy phase for the AT group. We assume that at some point, the size of the endpoint error exceeded the participant's self-defined tolerance for errors and caused them to modify the strategy. Unfortunately, we do not have a direct measure of strategy change. Examination of the learning profiles revealed considerable variability across individual participants (Figure 4A and Figure 5). This variability likely reflects multiple sources of noise, as well as instability in the use of a strategy. We obtained self-reports in a debriefing session at the end of the experiment. A few subjects in the AT and DAT groups reported adjusting their strategy such that they reached to a location between the cued target and aiming target, or that shifted to reach straight to the cued target.

At a minimum, multiple processes are required to capture this nonmonotonic learning function. In our initial modeling efforts, we fixed the strategy for the entire training process. Under this assumption, the system should exhibit drift that is equal in size to the rotation, an effect never observed. Thus, the final version of our model is a variant of a two-rate state space model [28], but with the two rates reflecting different error sources. As described above, adaptation of an implicit model is driven by the aiming error. In contrast, the strategy is adjusted on a trial-by-trial basis as a function of the current target error. Target errors are initially quite small and, thus have little effect on performance. However, as the target errors become large due to adaptation of the internal model, adjustments in the strategy are required to improve endpoint accuracy. Aiming to a new location resets the recalibration around a new setpoint. To reach a stable state, participants would need to progressively adjust their strategic aiming location to a point where aiming error and target error cancel each other out.

It is reasonable to assume that our manipulation of the availability of the aiming locations influenced the degree of certainty associated with the desired aiming location. When certainty is reduced, adaptation arising from the aiming error signal is slower, and in our two-process model, the level of adaptation achieved by the motor system is lowered. Moreover, the model does not predict that drift will reach 45°. The strategy is adjusted, reaching a point where it offsets the drift arising from adaptation of the internal model. The interplay of these two processes is complex (Figure 6D). With both occurring continuously during training, the system reaches a pseudo-equilibrium state at which additional changes to both processes becomes relatively small.

Linking the strategy adjustment to the target error signal offers a process-based approach to capture flexibility in strategy use. Our setpoint model captures this through the strategy adjustment parameter (F), a weighting term on target error. The NoAT group appears to give more weight to target error than the AT and DAT group. Interestingly, the modeling results indicate that the AT group showed more utilization of the target errors than the DAT group. We assume this arises because the AT group eventually offset the relatively large drift to restore on-target accuracy. In contrast, the DAT group never corrected for drift, suggesting that the weight given to target errors for this group was nearly zero.

It is important to highlight one difference in how we conceptualize changes in the rate of strategy adjustment (F) compared to changes in the rate of adaptation (B). Adjustments in a strategy can occur on very fast timescale; for example, once instructed, participants were able to immediately offset the full rotation. Variation in F refers to the rate at which participants change where to aim. In contrast, B reflects a gradual process, reflecting the rate of change in a system designed to reach a desired location.

### Alternative models of strategy change

In many sensorimotor adaptation tasks, variable learning rates are used to model the substantial variability observed in individual learning curves. In a similar manner, our setpoint model captures individual differences in strategy utilization by varying the strategy adjustment rate (F). Nonetheless, this formulation does not adequately capture the full range of behavior observed in the current study. In particular, this approach is insufficient to account for abrupt changes in performance. For example, the learning profile shown in Figure 5B suggests a categorical change in strategy. That is, the participant abandoned what was becoming an unacceptable strategy to search for a new strategy. Indeed, in a post-test interview, this participant reported changing the aiming location to a position halfway between the cued target and the aiming location.

An alternative approach to model strategy change could be derived from models of reinforcement learning [34]–[37]. In such models, participants explore different regions of a strategy space, attempting to quickly identify the policy that results in small target error. In our task, a shift in policy might occur when the rise in target error due to adaptation exceeds a threshold. That is, when a chosen action fails to achieve the predicted reward, a new strategy is adopted. This approach would provide a way to fit the data of the few participants who exhibited categorical-like changes in performance.

A reinforcement learning approach based on a discrete set of strategies is problematic with the current data set. At one extreme, one might suppose that such values could take on the locations of the aiming targets (e.g., 0° and 45°), and perhaps some intermediary points (e.g., 22.5°, the point halfway between two aiming targets). At the other extreme, the set might consist of a large set of values. Choosing a sparse set of potential actions will result in more abrupt changes in performance, while choosing a finer set of potential actions will allow for more gradual changes. Studies designed to explore reinforcement learning models generally use a limited set of choices and performance thus entails discrete shifts in behavior. In our task, reach direction spans a continuous space, and in fact, for most of our participants, the changes in performance were gradual. Future experiments that constrain the set of potential actions and manipulate reward may be better suited for employing a reinforcement learning perspective to explore strategy change.

Qualitative changes in performance may also indicate that the participants have fundamentally changed their conceptualization of the task. For example, rather than view the task goal as one involving reaching to targets, the participant may have switched to an orientation in which the task goal involved mastering a game in which the hand is a tool [38]–[40]. By this account, the initial drift would result from the operation of implicit adaptation of an internal model of the arm as described above. However, when this drift became too large, the participant switched to treating the task as a game, with the arm now conceptualized in a manner similar to how we view a computer mouse. Accurate performance now required learning the appropriate transformation between the movements of the tool and the task workspace. The error signal for this form of learning would no longer be based on the difference of predicted hand/object location and the feedback location; we are able to readily accept that the movement of a mouse-driven cursor results in feedback in an alternative workspace. Rather, the error signal here is the difference between the cued target location and the feedback location. An error signal of this form would not produce drift.

The reconceptualization hypothesis would predict that peak drift should equal or be greater than the aftereffect. This follows from the idea that adaptation of the internal model should cease at the time the task goal changes from reaching to tool mastery. Once the participant switches from learning about their arm to learning how to play the visuomotor game, then there the internal model would not continue to learn. The target error gains emphasis and the aiming error falls out. As such, the aftereffect should equal the drift value or be lower if there is some time-dependent decay of the adaptation effects [41].

While this hypothesis is plausible, there are also some limitations. First, it is important to keep in mind that in almost all adaptation studies, the only visual signals are the target location and a feedback cursor. Under such conditions, aftereffects are prominent, indicating adaptation of an internal model and not just learning a game. One would have to assume that tool conceptualization was more pronounced in the present study because of the strategic instructions. Second, our estimate of the aftereffect is actually larger than the peak drift for two of the three groups (Figure 4D). This observation, while at odds with the reconceptualization hypothesis, is consistent with the setpoint model. In our model, the aiming error signal will continue to modify the internal model even as strategy adjustments reduce target error. As such, the aftereffect, an estimator of implicit adaptation should be equal to or larger than peak drift.

While future research will be required to explore the mechanisms of strategy change, the current study advances our understanding of the interactions that arise between explicit, strategic processes and implicit, motor adaptation. Consistent with Mazzoni and Krakauer [9], the results make clear that strategies should not be viewed simply as representations that can facilitate implicit learning mechanisms. Rather, implicit learning mechanisms operate with a considerable degree of autonomy and, under certain conditions, can override the influence of an explicit strategy. Nonetheless, the benefits of strategic capabilities are also borne out in the present work. When implicit mechanisms go awry, a strategic system can confer the flexibility required to ensure task success.

## Methods

### Ethical statement

The study was conducted according to the principles expressed in the Declaration of Helsinki and the protocol was approved by the University's IRB. Thirty right-handed participants with no known neurological conditions were recruited from the University of California research participation pool. All participants provided informed consent prior to the start of the experiment.

### Experimental apparatus and procedures

The participant was seated in front of a table with her right hand comfortably positioned on a table surface. A horizontal, back-projection screen was positioned 48 cm above the table and a mirror was placed halfway between this screen and the table surface. The displays were presented via an overhead projector. By having the participant view the mirror, the stimuli appeared to be presented on the table surface. The mirror occluded vision of the hand; thus, feedback, when provided, was given in the form of a small red circular “cursor”. Movements were tracked by a 3D motion tracking system (miniBIRD, Ascension Technology, Burlington, VT, USA). A sensor was placed on the tip of the index finger, and position information was sampled at 138 Hz. The miniBirds have an approximate spatial resolution of 0.05 cm.

On each trial, the participant made a horizontal reaching movement to a visually displayed target, sliding their hand along the surface of the table. The target was defined by the appearance of a green circle at one of eight possible locations and the eight locations were separated by 45° on a virtual ring with a radius of 10 cm, centered on the starting position. The targets were not at cardinal directions, but started at 22.5° and increased in 45° steps. Participants were instructed to move quickly and were not provided with online visual feedback during the movement. Once the hand crossed the virtual target ring, a stationary red feedback cursor was displayed for 1000 ms. Subsequent to the feedback interval, the participant was visually guided back to the starting location. A white circle appeared, with the diameter corresponding to the distance of the hand from the starting position. The participant was trained to move so as to reduce the diameter of this circle. When the hand was within 10 pixels (8.8 mm) of the starting position, the circle changed to a cursor that the participant then moved into the start location. When this position had been maintained for 500 ms, the next target appeared. The target, start region, and feedback cursor were all 8 pixels (7 mm) in diameter.

Testing began with a familiarization block in which participant was trained to make rapid reaching movements from the start location toward the target location (Figure 2A). The participants were instructed that the task goal was to make the red feedback cursor appear as close as possible to the green target. We did not impose any constraint on movement amplitude other than that the movement had to span at least 10 cm. In addition to emphasizing the importance of directional accuracy, the participants were trained to complete the movement within 300 ms. Auditory feedback in the form of computerized voice that said “too slow”, was provided if the movement took longer than 300 ms. The quick movements were intended to minimize any within-movement control even though there was no online visual feedback provided. The familiarization block lasted 40 trials, five for each of the eight target locations.

Following the familiarization block, participants were trained to use a 45° clockwise (CW) strategy. Eight blue circles (termed “aiming target”— see below), indicating the possible target locations, were always visible during these trials; one of these turned green to indicate the target. The participants were instructed to aim to the neighboring CW blue circle (Figure 2B). The feedback cursor was veridical, appearing at the position of the hand when it crossed the virtual target ring. Thus, during this block, the participant attempted to align the feedback cursor with the blue circle that was 45° clockwise from the green target. This strategy-only block consisted of 40 trials.

The strategy-only block was followed by a 40-trial baseline block in which participants were instructed to reach directly towards the green target. The feedback remained veridical and thus, the participant's goal was to align the feedback cursor with the green target.

Following these 40 trials, a visuomotor rotation was introduced without warning. For these trials, the position of the feedback cursor was shifted −45° (CCW rotation) from the actual hand position (Figure 2C), and correspondingly, induced substantial endpoint error. After two such movements, the participants were told that they could minimize their error by adopting an explicit “corrective” strategy in which they aimed 45° in the CW direction from the target (Figure 2D). The participants were instructed to employ this strategy and testing continued without further change for the next 320 trials (40 movements/target).

At the end of this rotation (plus strategy) block, there was a brief pause so that the experimenter could instruct the participants the rotation would no longer be present and that they should resume moving directly to the cued target location. For the next eight trials (1/target location), no endpoint feedback was provided. The purpose of this short block was to quantify the aftereffects of the rotation training, while not inducing any learning based on visual errors [28].

The experiment concluded with a washout block that was identical to the baseline block. The rotation remained off and participants were reminded to continue reaching towards the green target. The feedback cursor was again visible, now providing an error in terms of the distance between this cursor and the green target.

There was a short temporal delay (less than 1 minute) between the blocks so that the experimenter could load the new block.

The participants were divided into three experimental groups. The only difference between the groups was the status of the blue circles, the visual landmarks that provide an aiming target during the strategy-only and rotation blocks. In the Aiming-Target group (AT group; 4 Female/6 Male, ages 19–25), the blue circles were present throughout the experiment. In the No Aiming-Target group (NoAT group; 5 Female/5 Male, ages 18–28), the blue circles were never presented except for the strategy-only practice block. In this block, the blue circles were presented on half of the trials to assist the participants in learning where 45° was in relation to the cued, green target. During the other blocks (familiarization, baseline, rotation, and washout blocks), the blue circles were absent at all times.

The third group was the Disappearing Aiming-Target group (DAT group; 5 Female/5 Male, ages 19–23). For these participants, the blue circles were presented at the start of the trial, followed shortly by a green target at one of the locations. The blue circles remained visible until the hand had been displaced 1 cm from the starting position (approximately 30 ms into the movement) at which point they disappeared. Thus, the aiming targets were not visible when the feedback cursor appeared. As in the other conditions, the green target remained on the screen until the end of the feedback interval. We opted to use blue circles on a black background as the visual landmarks to minimize visual aftereffects for the DAT group.

### Movement analysis

Kinematic information was analyzed with Matlab (MathWorks, Natick, MA). Movement duration was defined as the interval from when the hand was 1 cm from the start position until it passed through the virtual target ring (10 cm radius). We determined the heading of the hand at the point of intersection and used this to compute the endpoint hand angle, defined as the difference between this heading and a straight line connecting the starting position and the target (green circle except for the strategy-only block). When there was no rotation, the target error was identical to the endpoint hand angle. When the rotation was present, the target error was the endpoint hand angle plus 45°. The angular endpoint error was used to infer the motor plan (plus noise) since the movements were made without on-line feedback and at a speed that minimized corrective movements. Since there was a substantial difference between groups in terms of drift, we measured the aftereffect relative to the target location.

For the analyses of movement accuracy, movements within each block were averaged over 10-trial bins. However, we did not bin the first two movements when the rotation was first introduced (pre-strategy), nor did we bin the first two movements after the strategy was introduced. Rather, these two movement pairs were averaged separately to quantify error introduced by the rotation and the initial success of the participant using the strategy, respectively.

A key dependent measure in this study is the magnitude of the drift exhibited during the rotation block. Estimating peak drift is difficult, not only because of noise in performance, but also because some participants exhibited non-monotonic drift functions. To minimize these problems, we used a boostrapping [42] method to estimate peak drift. Using the group averaged data, we created bins of 10 movements each and then identified the bin with the largest angular deviation. The group averaged data was recompiled 1000 times by randomly resampling with replacement from the participant pool. The estimate of the time of the peak drift was chosen as the movement number in the middle of this 10-movement bin.

We used a similar method to compute the aftereffect. Here we focused exclusively on the first 8 trials following the end of the strategy plus rotation phase, trials in which no visual feedback was provided. The bootstrapping method here produces only a slightly different estimate of the aftereffect compared to a simple averaging across the observed data from these 8 trials.

To quantify the deadaptation rate during the washout phase, we fit an exponential function [26] to the time series of target endpoint errors. Specifically, we bootstrapped the washout data from each group to provide an estimate of the exponential decay rate. We compared these rates to determine if there was a difference in the rate of deadaptation.

To statistically evaluate the results of the bootstrapping procedures, the mean statistics of each resampled iteration were calculated and then used to determine p values [29], [42]. All statistical analyses were performed in Matlab. For the analyses that did not involve bootstrapping, we report the degrees of freedom for the F-values when performing ANOVAs across groups and t-values when performing t-tests within groups.

Occasionally participants did not move to the cued, green target (on baseline and washout blocks), mistakenly implemented the strategy in the wrong direction (i.e., went CCW instead of CW on rotation blocks), or moved to a location far from the target. We eliminated trials in which the movement heading was more than three standard deviations from the mean for that block. This resulted in an average removal of less than 1% of the movements per participant and the number of such erroneous movements was similar across the three groups (F_2,27_ = 1.58, p = 0.22).

### Modeling

The Nelder–Mead method or simplex method [43], implemented in Matlab as fminsearch, was used to fit the data from the baseline, rotation, stop-strategy, and washout blocks. We did not fit the data from the familiarization block and strategy-only block. The instruction to use the strategy was implemented by setting the value of s to 45 at the start of the strategy+rotation block. The value was reset to 0 at the start of the washout block. While the simplex method can be sensitive to initial conditions, we obtained similar estimates of the parameters (within the confidence intervals of those parameters) with different starting values with the current data sets. Thus, the same initial conditions (values of zero for all parameters) were used for each participant. The goodness of fit was measured by the root mean square error (rms) and Pearson's correlation coefficient (r) between the output of the model for endpoint hand angle and the participant's endpoint hand angle. Custom software was written to bound the parameters between 0 and 1. The sign of the parameters is dependent on the convention we used for the target errors: CCW to the target was negative and CW was positive. The equations were adjusted to make all the parameters positive. The parameter K was bound from 0.1 to 1 for all groups because we found that the simplex method sometimes reached a local minimum of K = 0 for the NoAT group (approximately 10% of fits). The data from the NoAT group is more difficult to fit because of the absence of drift and relatively small aftereffect.

A, B, and E, the parameters characterizing the internal model memory, adaptation gain, and strategy memory, were fit for all the groups collectively. K the parameter characterizing the availability of the aiming target (strategic aiming location) and F, the influence of target errors, were estimated separately for each group through bootstrapping. The group's averaged data was computed by resampling with replacement the participant pool, repeating this 1000 times, and fitting the setpoint model (Eqns 8–11) to each resampled average.
